# Dressed Gain from the Parametrically Amplified Four-Wave Mixing Process in an Atomic Vapor

**DOI:** 10.1038/srep15058

**Published:** 2015-10-14

**Authors:** Zhaoyang Zhang, Feng Wen, Junling Che, Dan Zhang, Changbiao Li, Yanpeng Zhang, Min Xiao

**Affiliations:** 1 Key Laboratory for Physical Electronics and Devices of the Ministry of Education & Shaanxi Key Lab of Information Photonic Technique, Xi’an Jiaotong University, Xi’an 710049, China; 2 Department of Physics, University of Arkansas, Fayetteville, Arkansas 72701, USA & National Laboratory of Solid State Microstructures and Department of Physics, Nanjing University, Nanjing 210093, China

## Abstract

With a forward cone emitting from the strong pump laser in a thermal rubidium atomic vapor, we investigate the non-degenerate parametrically amplified four-wave mixing (PA-FWM) process with dressing effects in a three-level “double-Λ” configuration both theoretically and experimentally. By seeding a weak probe field into the Stokes or anti-Stokes channel of the FWM, the gain processes are generated in the bright twin beams which are called conjugate and probe beams, respectively. However, the strong dressing effect of the pump beam will dramatically affect the gain factors both in the probe and conjugate channels, and can inevitably impose an influence on the quantum effects such as entangled degree and the quantum noise reduction between the two channels. We systematically investigate the intensity evolution of the dressed gain processes by manipulating the atomic density, the Rabi frequency and the frequency detuning. Such dressing effects are also visually evidenced by the observation of Autler-Townes splitting of the gain peaks. The investigation can contribute to the development of quantum information processing and quantum communications.

The parametric four-wave mixing (FWM) process based on atomic coherence^1^,^2^,^3^ plays important roles in the squeezed and entangled states of optical fields^4^,^5^,^6^,^7^ as well as the high-order cascaded nonlinear optical process^8^,^9^. For the cascaded-nonlinearity process in an atomic system, the FWM process can serve as a low-noise linear phase-insensitive or phase-sensitive amplifier^10^,^11^ with easily adjustable parameters. Moreover, with the introduction of the squeezed state based on FWM process^12^,^13^, the progresses in the squeezing of light involve in various applications such as the generation of the multi-spatial-mode entangled beams^14^,^15^,^16^ and continuous-variable quantum images processing^17^,^18^,^19^. In recent years, the non-degenerate FWM process driven by a strong pump beam in a coherent “double-Λ” atomic system without the disturbance of a cavity is demonstrated to generate strong relative intensity squeezing between the bright twin beams named as probe and conjugate^20^,^21^. Also, the diamond-type four-level configuration^22^,^23^,^24^,^25^ with a stable ground state, two intermediate levels, and an upper state in an atomic system is also developed to study non-degenerated FWM process at the wavelength for telecommunication^26^,^27^. Compared with the diamond-type configuration (5*S*_1/2_, 5*P*_1/2_, 5*P*_3/2_, and 6*S*_1/2_) in a rubidium vapor, the “double-Λ” system can be more efficient due to the low requirement on phase matching condition (PMC). For the FWM process characterized as large nonlinearity at near resonance and reduced the excess noise^15^ in the “double-Λ” system, the probe and conjugate beams can be both amplified and then induce the generation of a pair of strongly correlated Stokes and anti-Stokes fields^28^ with high efficiency, which can produce squeezed states with narrow bandwidth and low frequency^20^,^29^. Actually, the parametrically amplified factor in the FWM process can directly determine the squeezing level of the two bright beams^15^,^17^.

In this article, we demonstrate the parametrically amplified FWM (PA-FWM) process with dressing effects in an off-resonant “double-Λ” arrangement by establishing a forward cone^30^ in a thermal rubidium atomic vapor both theoretically and experimentally. On one hand, with the weak probe beam seeded into the Stokes or anti-Stokes channel in the FWM process generated from the strong pump beam, the gain processes can be obtained in both the conjugate and probe channels. On the other hand, with the pump filed considered as a coupling or dressing field, the classical electromagnetically induced absorption (EIA)^31^ is also observed in the probe channel. As a result, the EIA effect can interact and compete with the gain process in the probe field, which can inevitably render the change of squeezing state as well as the amplification factor. Such interaction and competition are investigated by the signal intensity evolution dependences of the dressed gain on the atomic density, the Rabi frequency and the frequency detuning of the pump laser. As a consequence, we can control quantum effects as well as the amplification factor in the FWM process by easily manipulating the corresponding parameters. The fundamental study on the dressed gain of the bright twin beams can be effectively conducive to the development of quantum information processing such as entangled images and the generation of correlated photons source as well as squeezing states.

## Results

**Figure 1:**
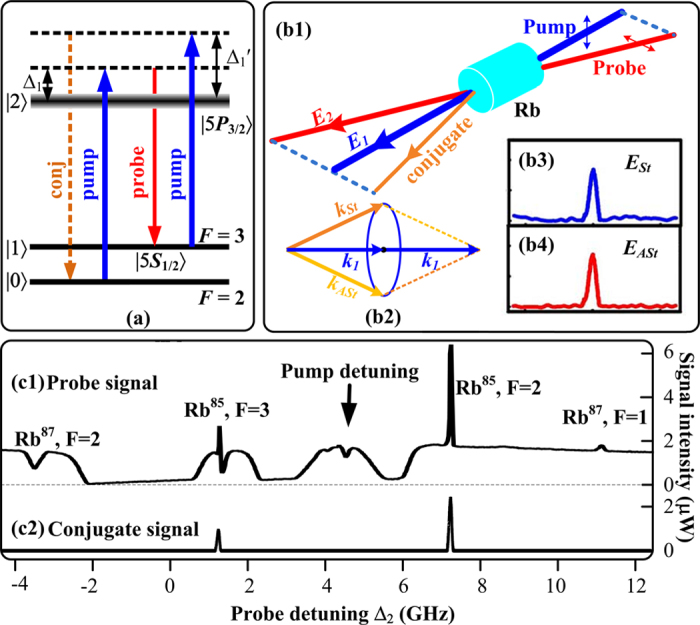
(**a**) Energy-level diagram for the double-Λ configuration in ^85^Rb atoms. (**b1**) Spatial beams alignment for the PA-FWM process. (**b2**) Phase-matching geometrical diagram of the PA-FWM process. (**b3**) Measured Stokes field ***E***_*St*_ and (**b4**) anti-Stokes field ***E***_*ASt*_ versus the pump frequency in the FWM process. (**c1**,**c2**) Measured probe transmission signal and corresponding conjugate signal versus the probe frequency detuning Δ_2_, respectively.

A three-level “double-Λ” type ^85^Rb atomic system, consisting of two hyperfine states F = 3 (|0〉) and F = 2 (|3〉) of the ground state 5S_1/2_ and an excited state 5P_3/2_ (|1〉), is used to generate the PA-FWM process. Two laser beams derived from a Ti:sapphire laser and an external cavity diode laser (ECDL) are coupled into the corresponding transitions as shown in Fig. 1(a). The spatial beams alignment is shown in Fig. 1(b). With the laser frequency tuned to the *D*2 line transition of rubidium atoms, the strong pump beam ***E***_1_ (frequency *ω*_1_, wave vector ***k***_1_, Rabi frequency *G*_1_, vertical polarization) up to 500 mW is coupled into the cell by a polarizing beam splitter (PBS). The weak probe beam ***E***_2_ (*ω*_2_, ***k***_2_, *G*_2,_ horizontal polarization) with approximately 100 *μ*W propagates in the same direction of ***E***_1_ with an angle of 0.26° and is detected by a branch of a balanced homodyne photodiode detector. The generated conjugate signal that can establish the coherence between the two ground states |0〉 and |1〉 co-propagates with***E***_2_ symmetrically with respect to ***E***_1_ and is received by the other branch of the balanced detector.

With the frequency detuning of ***E***_1_ tuned far away from the resonance, the FWM process will occur in the “double-Λ” configuration, which can generate the Stokes field ***E***_*St*_ and anti-Stokes field ***E***_*ASt*_ (satisfying the PMCs ***k***_*St*_ = 2***k***_1_ − ***k***_*ASt*_ and ***k***_*ASt*_ = 2***k***_1_ − ***k***_*St*_, respectively) on the forward cone shown in Fig. 1(b2). Here the detuning Δ_*i*_ = Ω_*i*_ − *ω*_*i*_ is defined as the difference between the resonant transition frequency Ω_*i*_ and the laser frequency *ω*_*i*_ of ***E***_*i*_. The detected ***E***_*St*_ and ***E***_*ASt*_ signals (via perturbation chains 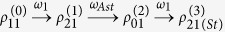 and 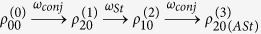 by means of the Liouville pathway^32^) are shown in Fig. 1(b3,b4), respectively. Here the corresponding third-order density matrix elements 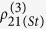
and 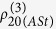
can be described as


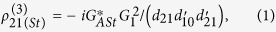



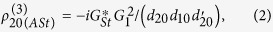


where *G*_*i*_ = *μ*_*ij*_*E*_*ij*_/*ħ* (*i, j* = 1, 2, *conj*, *St*, *ASt*) is the Rabi frequency between levels |*i*〉 ↔ |*j*〉, and *μ*_*ij*_ is the dipole momentum; *d*_20_ = *γ*_20_ + *i*Δ_1_, *d*_21_ = *γ*_21_ + *i*Δ_1_, *d*_10_ = *γ*_10_ + *i*(Δ_1_ − Δ_*St*_), 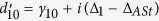, 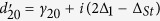, 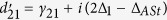; Γ_*ij*_ is the nature decay rate between levels |*i*〉 and |*j*〉 and *γ*_*ij*_ = (Γ_*i*_ + Γ_*j*_)/2 is the decoherence rate between levels |*i*〉 and |*j*〉.

The presence of the weak probe beam ***E***_2_ can be viewed as being injected into the Stokes or anti-Stokes port of the FWM process, and the injection will serve as an optical parametric amplification (OPA) process (with PMC ***k***_*ASt*_ = 2***k***_1_ − ***k***_2_ or ***k***_*St*_ = 2***k***_1_ − ***k***_2_) assisted by the cascaded nonlinear process. With the perturbation chains rewritten as 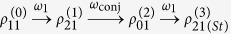 and 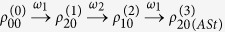, Eqs (1 and 2) should be modified as


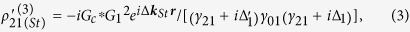



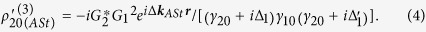


As a result, the input probe beam with a intensity of *P*_2_ is amplified to produce an output probe with a intensity of *gP*_2_ and an output conjugate with a intensity of (*g*–1)*P*_2_. See **Methods** for the theoretical derivations of the gain factor *g*. Taking the dressing effects of ***E***_1_ and ***E***_2_ into consideration, the expression for the Stokes and conjugate anti-Stokes fields should be written as


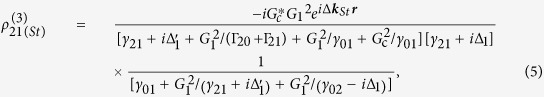



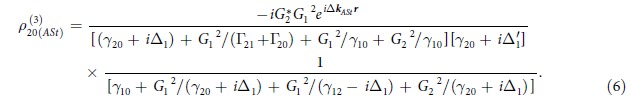


Also, with ***E***_2_ and ***E***_1_ viewed as probe and coupling fields, respectively, the first-order density matrix 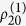 for the probe transmission signal with dressing effect is


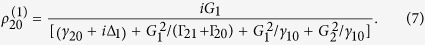


Consequently, the intensity *I*_*p*_ of the probe signal with gain and dressing effect can be expressed by 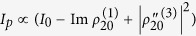 and 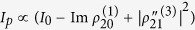 for transitions *F* = 3 → *F*′ and *F* = 2 → *F*′, respectively, where *I*_0_ is the intensity of the probe field without Doppler absorption.

By experimentally scanning the probe detuning over 16 GHz across the *D*2 line, we obtain the featured transmission signal and corresponding conjugate signal as shown in Fig. 1(c1,c2), respectively. The observed character in the probe channel can be attributed to Raman absorption, EIA effect, parametrical amplification process, and the combined dressing effects. The absorptive dip at Δ_2_ = −3.5 GHz has a linewidth of 278.5 MHz and is caused by Raman absorption on ^87^Rb, *F* = 2 → *F*′. The dip at Δ_2_ = 4.5 GHz indicates the detuning of the pump field. The most prominent points are the Stokes and anti-Stokes gain peaks at the ^85^Rb ground-state hyperfine states (Δ_2_ = 1.2 GHz and 7.2 GHz, respectively). According to the experimental condition, there exists 6 GHz frequency gap between the Stokes and anti-Stokes signals in the probe channel. The seeding process can also generate the anti-Stokes and Stokes peaks in the conjugate channel corresponding to the Stokes and anti-Stokes in the probe channel, respectively. The Stokes (*F* = 3 → *F*′) in the probe channel and the corresponding anti-Stokes in the conjugate channel are strongly correlated, so do the anti-Stoke (*F* = 2 → *F*′) of the probe and Stokes of the conjugate.

### The Stokes and anti-Stokes gain processes in the probe channel

**Figure 2:**
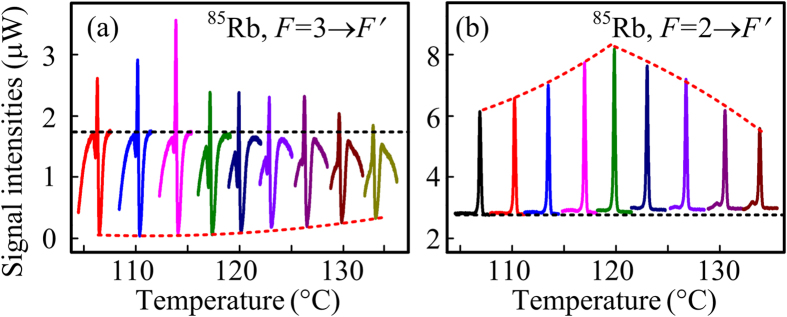
Intensity evolutions of the Stokes (a) and anti-stokes (b) gain peaks in the probe channel versus Δ_2_ by increasing the ^85^Rb atomic density. The atomic density is about 3 × 10^13^ cm^−3^ at 120 °C. The dotted lines are guides for eyes.

Figure 2(a,b) show the Stokes (^85^Rb, *F* = 3 → *F*′ transition) and anti-Stokes (^85^Rb, *F* = 2 → *F*′ transition) signal evolutions in the probe channel by varying the atomic intensity, respectively. The single Stokes signal profile in Fig. 2(a) obviously consists of an EIA dip with a full width at half maximum (FWHM) of 101.7 MHz and a gain peak with a FWHM of 32.4 MHz. Here, the sharp gain peak is caused by seeding ***E***_1_ into the Stokes port of the FWM process, and the gain intensity is related to the generated photon number described by Eq. (11) in **Methods** part. The EIA dip reveals the dressing effects when ***E***_1_ and ***E***_2_ beams are viewed as coupling and probe fields, respectively. The single anti-Stokes peak with a FWHM of 50.8 MHz in Fig. 2(b) also results from the injection of ***E***_2_ beam, which can be attributed to the amplification process in Eq. (10) related to *F* = 2 → *F*′ transition.

The measured intensity of the Stokes gain by increasing the atomic density in Fig. 2(a) is first getting larger and then smaller, which demonstrates the competition between the atomic dynamics process and the propagation of the FWM process. At the lower atomic density (105 °C–115 °C), the gain directly proportional to the atomic density is dominated by the increase of the thermal motion of the atoms, while the absorption of FWM is small. With the density continuing to grow (115 °C–125 °C), the atomic dynamics process and the absorption can achieve a balance and the signal intensity remains almost unchanged. When the temperature is over 125 °C, the decrease of the gain can be attributed to the fact that the absorption of the FWM during its propagation is increasing while the atomic motion gets saturated at high atomic density, namely, the absorption can be dominant. The varying tendency of the anti-Stokes peak in Fig. 2(b) is similar to that of the Stokes peak described previously. The EIA depth in Fig. 2(a) described with term 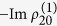 can experience growing, unchanged and getting smaller by increasing the atomic density. The first growth and saturation can be attributed to the dominated dressing effects caused by *G*_2_ and *G*_1_ and the subsequent weakening is similarly due to the strengthening of the Doppler effect.

### The amplified Stokes and anti-Stokes signals in the conjugate and probe channels

**Figure 3:**
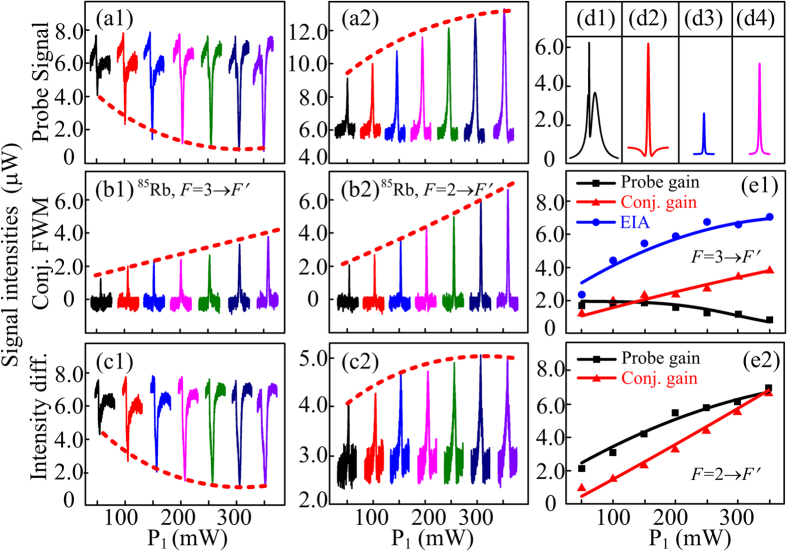
Measured probe and corresponding conjugate signals versus Δ_2_ at different pump power. (**a1**,**a2**) Intensity evolutions of the Stokes and anti-Stokes gain peaks at probe channel versus Δ_2_ by increasing the power of ***E***_1_, respectively. (**b1**,**b2**) Phase conjugate signals corresponding to (**a1**,**a2**), respectively. (**c1**,**c2**) Intensity difference between the probe and conjugate signals related to *F* = 3 → *F*′ and *F* = 2 → *F*′, respectively, by use of the homodyne detector. The dotted lines are guides for eyes. (**d1**,**d4**) Simulated lineshapes correspond to the signals in (**a1**,**a2**,**b1**,**b2**), respectively. The theoretical simulations in (**e1**,**e2**) agree well with the experimental observations. The squares, triangles and dots represent the original experimental observations of corresponding signals and the solid curves are the theoretical predictions.

Figure 3(a1,a2) are the dressed Stokes and Anti-Stokes signals, respectively, from the probe output by growing the power of pump field with the conjugate detection branch blocked. For the Stokes signal, the peak represents the gain intensity and the dip is the classical EIA intensity. According to Eq. (3), the gain peak should increase with the square of pump Rabi frequency *G*_1_. At small ***E***_1_ power (*P*_1_), the gain can increase with *G*_1_, which is observed in Fig. 3(a1) with *P*_1_ changing from 30 mW to 90 mW. However, due to the strong dressing effects caused by denominator terms 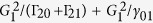 and 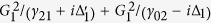 in Eq. (5), the peak can then be suppressed when *P*_1_ is getting larger. Also, the EIA dip can be deepened due to the increase of *G*_1_ as described by Eq. (7). For the anti-Stokes in Fig. 3(a2), the dressing effects are weak with Δ_1_ being far away from the resonance. Consequently, the anti-Stokes gain described by Eq. (4) can increase with *G*_1_ and the gain can also be much stronger than that in Fig. 3(a1).

The anti-Stokes and Stokes signals that are conjugate to the two gain peaks in the probe channel are shown in Fig. 3(b1,b2), respectively. Physically, the two pairs of generated Stokes and anti-Stokes signals by scanning the probe detuning are correlated in frequency and space, so the measured anti-Stokes and Stokes signals in the conjugate channel should behave similarly to the corresponding signals in the probe channel. However, due to the absence of directly dressing effect, both the intensities of Stokes and anti-Stokes in the conjugate channel can grow with *G*_1_, which are depicted in Fig. 3(b1,b2) and can be explained by Eqs (3 and 4), respectively. With the twin branches of the detector turned on, Fig. 3(c1,c2) are the measured intensity difference evolutions on *F* = 3 → *F*′ and *F* = 2 → *F*′ transitions with different *G*_1_, respectively. Figure 3(d1,d2,d3, and d4) are the simulated lineshapes for the Stokes and anti-Stokes in the probe channel and anti-Stokes and Stokes in the conjugate channel. The theoretical curves in Fig. 3(e1,e2) agree well with the experimental results.

**Figure 4:**
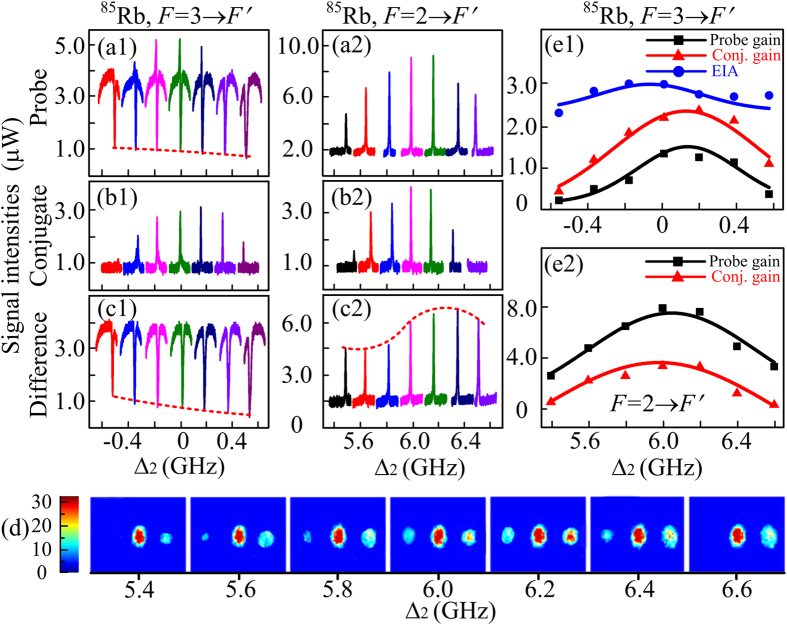
Measured probe and corresponding conjugate signals versus Δ_2_ at different Δ_1_. (**a1**,**a2**) Stokes and corresponding anti-Stokes gain peaks in the seeding channel, respectively. (**b1**,**b2**) Conjugate FWM signals corresponding to (**a1**,**a2**), respectively. (**c1**,**c2**) Measured intensity difference between the probe and corresponding FWM signals related to *F* = 3 → *F*′ and *F* = 2 → *F*′, respectively. The dotted lines are guides for eyes. (**d**) Measured images of the probe and conjugate signals corresponding to (**a2**,**b2**), respectively. (**e1**,**e2**) Intensity dependences on pump detuning corresponding to *F* = 3 → *F*′ and *F* = 2 → *F*′ transitions, respectively. The squares, triangles and dots are the original experimental observations of corresponding signals and the solid curves are the theoretical predictions.

Figure 4(a1,a2) are the Stokes and anti-Stokes measured from the probe channel at different Δ_1_, respectively. Since the horizontal positions of the two gain peaks on the probe signal can move with the pump detuning changing, the probe detuning values Δ_2_ in Fig. 4 can directly depict the change of Δ_1_, which can also be consistent with the coordinate probe detuning shown in Fig. 1(c1). The peaks in Fig. 4(a,b) should be the highest near Δ_2_ = 0 and Δ_2_ = 6 GHz, respectively, due to the improved nonlinearity caused by the strong coherence in the FWM process at resonance. Similarly, the EIA and dressing effects can also be strengthened, which in turn can impose stronger suppression on the gain peaks. As a result of the interaction, the gain peak can have the almost unchanged intensity with Δ_1_ varying from Δ_2_ = −200 MHz to 200 MHz. The optimum choice of pump detuning Δ_1_ should be a balance between the increasing of gain and reduction of the dressing effect as well as the probe loss with Δ_1_ tuned close to the atomic line. By blocking the probe detection branch, the corresponding anti-Stokes and Stokes are received from the non-injected channel and demonstrated in Fig. 4(b1,b2), respectively. According to Eqs (5 and 6), the third-order polarization 
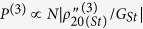
or 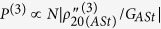 at Δ_1_>0 decreases with the increase of Δ_1_ with *G*_1_ ≫ *G*_2_ and *G*_1_ ≫ *G*_c_ considered, which can also interpret the height evolutions of the observed gain peaks well. The intensity difference changes between the probe and conjugate signals at different Δ_1_ are shown in Fig. 4(c1,c2). Figure 4(d) provides the images of the probe and conjugate beam for *F* = 2 → *F*′ transition at different Δ_1_. The right image and left image in each picture are the probe signal and conjugate signal and visually demonstrate the signal intensity evolutions corresponding to Fig. 4(a2,b2), respectively. The strong signal image in the middle of each picture with almost unchanged intensity are the residual transmission of the pump beam (vertical polarization) after the polarizing beam splitter (PBS). Figure 4(e1,e2) are corresponding theoretical predictions and coincidence with the experimental observations.

## Discussion

**Figure 5:**
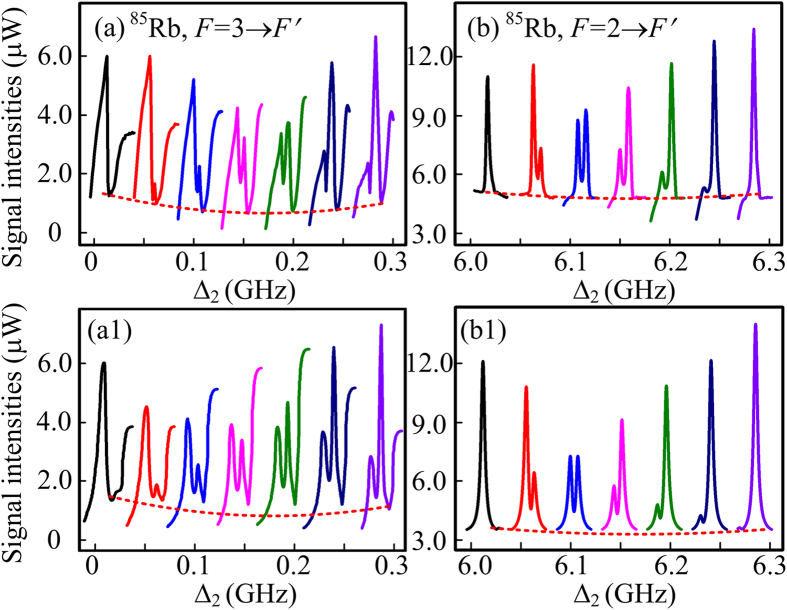
AT splitting of the Stokes (a) and anti-stokes (b) gain peaks in the probe channel versus Δ_2_ by varying the pump detuning Δ_1_. (**a1**,**b1**) Theoretical predictions corresponding to (**a**,**b**), respectively. The dotted lines are guides for eyes.

So far, we have presented the PA-FWM process with dressing effects assisted by the cascaded-nonlinearity process in a “double-Λ” ^85^Rb atomic system both theoretically and experimentally. With the parametrically FWM process established, we can obtain bright twin beams with gain by seeding a weak probe beam into the Stokes or anti-Stokes channel of the FWM process. Also, the dressing effects of the strong pump, the weak probe and the generated conjugate fields can impose influences on the probe gain and the conjugate gain. As an extension to our discussion, we present another powerful evidence for the dressing effects on the gain processes. To be specific, the observed Autler-Townes (AT) splitting^27^ on the gain peaks shown in Fig. 5 can be attributed to the interaction between the gain processes and the combined dressing effects.

Figure 5(a,b) are the observed AT splitting of the gain processes on ^85^Rb, *F* = 3 → *F*′ and ^85^Rb, *F* = 2 → *F*′, respectively, by varying the detuning of pump field. Similarly to Fig. 4, the probe detuning Δ_2_ in Fig. 5 can also reflect the pump detuning Δ_1_. When the single-peak gain in Fig. 5 is split into two peaks, the AT splitting caused by the combined dressing effects is obtained. The AT splitting effect in Fig. 5(a) firstly appears at Δ_2_ = 0.05 GHz and then the intensities of left and right peaks can change with Δ_1_, which means that the strong pump laser starts to dress the energy levels |1〉 and |2〉 to create corresponding primary dressing states^33^ |1+〉 & |1−〉 and |2+〉 & |2−〉, respectively. The two dressing state effects can be described by perturbation chains 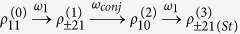 and 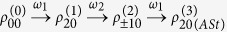, respectively. Such dressing effect with the participation of the two third-order nonlinear processes can dress the involved energy levels, which can then affect the third-order Stokes and anti-Stokes signals themselves. The measured left and right peaks in the Stokes gain in Fig. 5(a) correspond to the dress states |2+〉 and |2−〉, respectively. With Δ_1_ tuned close to the dress state |2+〉, the left peak is much higher than the right one. Similarly, the right peak can be enhanced with Δ_1_ tuned close to |2−〉. Considering the parallel generation process for the anti-Stokes, the dressing states |1+〉 and |1−〉 can also explain the evolution from single peak to double peaks in Fig. 5(b) well. The theoretical simulation results in Fig. 5(a1,b1) agree well with the observed results in Fig. 5(a,b), respectively.

With the interaction between the dressing state effects and gain processes systematically investigated by the intensity dependences of the dressed gain on the atomic density, the Rabi frequency and the frequency detuning, we can control the amplification factor in the FWM process by easily manipulating the corresponding parameters. Furthermore, the current study on controlling the dressed gain of the bright twin beams can have contribution to the development of entangled images and the generation of correlated photons source as well as squeezing states.

## Methods

### Experimental setup

We use two light beams from a continuous-wave Ti:sapphire laser and an ECDL to couple the three-level “double-Λ” rubidium atomic system. The strong pump laser beam ***E***_1_ and the weak probe beam ***E***_2_ have diameters of 0.7 mm and 0.4 mm respectively. The pump beam with vertical polarization is first coupled into the cell by use of a polarizing beam splitter (PBS) and then separated from the detection path via another PBS cube after the cell. The probe beam propagates in the same direction of ***E***_1_ with an angle of 0.26° and intersects with the pump beam at the center inside the cell with natural abundance. The 10 mm long rubidium cell with Brewster’s angle is wrapped by *μ*-metal and heated by the heater tape. The weak probe and the generated conjugate beam are received by a balanced phtotodetector with 10^5^ V/A transimpedance gain and ≈82% quantum efficiency.

### Theoretical models for the gain factors

For the case of the FWM process without seeding, the generated twin-photon numbers of the Stokes and anti-Stokes channels that are proportional to the signal intensity can be shown as


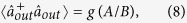



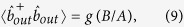


where 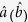 is the boson-creation operator of *E*_*St*_ (*E*_*ASt*_), and 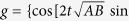

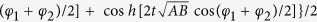
 is the gain with the modulus *A* and *B* (phase angles *φ*_1_ and *φ*_2_) defined in 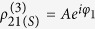 and 
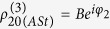
 for *E*_*St*_ and *E*_*ASt*_, respectively. Here the amplification factors are the same for the two output ports of the amplifier without seeding. When the weak probe field is injected into the Stokes port of the FWM process, it can amplify the seeded signal in an appropriate condition. The photon numbers of the output Stokes and anti-Stokes fields in the amplification process with injection are described as


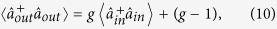



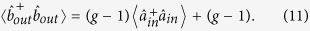


## Additional Information

**How to cite this article**: Zhang, Z. *et al.* Dressed Gain from the Parametrically Amplified Four-Wave Mixing Process in an Atomic Vapor. *Sci. Rep.* **5**, 15058; doi: 10.1038/srep15058 (2015).

## Supplementary Material

Supplementary Information
