# Supplementary material for: Dressed Gain from the Parametrically Amplified Four-Wave Mixing Process in an Atomic Vapor
Source: Sci Rep. 2015 Oct 14;5:15058. doi: 10.1038/srep15058 (PMC4604462; doi:10.1038/srep15058)
Supplement: Supplementary Information [file srep15058-s1.doc]

**Supplementary Information for “Dressed Gain from the Parametrically Amplified Four-Wave Mixing Process in an Atomic Vapor”**

Zhaoyang Zhang^1^, Feng Wen^1^, Junling Che^1^, Dan Zhang^1^, Changbiao Li^1^, Yanpeng Zhang^1^* and Min Xiao^2^

^1^Key Laboratory for Physical Electronics and Devices of the Ministry of Education & Shaanxi Key Lab of Information Photonic Technique, Xi’an Jiaotong University, Xi’an 710049, China

^2^Department of Physics, University of Arkansas, Fayetteville, Arkansas 72701, USA & National Laboratory of Solid State Microstructures and Department of Physics, Nanjing University, Nanjing 210093, China

*Corresponding authors: [ypzhang@mail.xjtu.edu.cn](mailto:ypzhang@mail.xjtu.edu.cn)

**The Supplementary Material mainly provides the derivations of the first- and third-order density matrix elements ($\rho_{20}^{(1)}$,**$\rho_{20(ASt)}^{(3)}$**and$\rho_{21(St)}^{(3)}$) in the manuscript.**

**1. The First- and Third-Order Density Matrix Elements $\rho_{20}^{(1)}$ and** $\rho_{20(ASt)}^{(3)}$

Considering the time-dependent Schrödinger equation, and using a perturbation expansion and the rotating-wave approximation, the density-matrix equations for the three-level “double-Λ” type atomic system are given as

(S1a)

(S1b)

(S1c)

(S1d)

(S1e)

where the detuning Δ*_i_*=Ω*_i_*−*ω_i_* is defined as the difference between the resonant transition frequency Ω*_i_* and the laser frequency *ω_i_* of ***E****_i_*; Γ*_ij_* is the nature decay rate between levels |*i*〉 and |*j*〉 and *γ_ij_*=(Γ*_i_*+Γ*_j_*)/2 is the decoherence rate; *G_i_*=*μ_ij_E_ij_*/*ħ* (*i, j*=1, 2…) is the Rabi frequency between |*i*〉↔|*j*〉, and *μ_ij_* is the dipole momentum.

With the weak probe beam ***E***_2_ injected into the anti-Stokes port of the FWM process, the ***E****_ASt_* signal can be described via perturbation chain. For the first process, a ground state ($\rho_{00}^{(0)}\text{+}\rho_{11}^{(0)}\text{=1}$) particle absorb a probe photon and transits to$\rho_{20}^{(1)}$(level |2〉). By solving Eq. (S1a) under the weak field approximation ($\tilde{\rho}_{10}^{(r-1)}\approx\tilde{\rho}_{22}^{(r-1)}\approx0$) and the steady state approximation ($\partial\tilde{\rho}_{20}^{(r)}/\partial t=0$), we can obtain, namely,

(S2a)

Similarly for the second step expressed as, we solve Eq. (S1b) with the approximation of weak field and steady state considered, we can obtain, namely,

(S2b)

For the third step expressed as, according to Eq. (S1d) under the approximation of weak field and steady state, is obtained. So we have

(S2c)

Substitute Eq. (S2b) into Eq. (S2c), then according to Eq. (S2a) and the ground state approximation$\rho_{00}^{\left(0\right)}\text{+}\rho_{11}^{\left(0\right)}\text{=}1$, and we can get

$\rho_{20(St)}^{(3)}=-iG_{2}^{*}G_{1}^{2}e^{i(2\mathbf{k}_{1}-\mathbf{k}_{2})\cdot\mathbf{r}}/\left[(\gamma_{20}\text{+}i\Delta_{1})\gamma_{10}(\gamma_{20}+i\Delta'_{1})\right]$ (S2d)

With the dressing effects of ***E***_1_ and ***E***_2_ considered, the perturbation chain then can be written as the dressed perturbation chain: . During the first step, according to (S1a), (S1b) and (S1c) under steady state approximation，the coupling equations can be obtained as

(S3a)

(S3b)

(S3c)

By solving Eqs. (S3a)-(S3c) with $\rho_{00}^{(0)}\approx1$，$\rho_{11}^{(0)}\approx0$and$\rho_{22}^{(0)}\approx0$taken into consideration，we have

$\rho_{20}^{(1)}=\frac{iG_{1}e^{i\mathbf{k}_{1}\cdot\mathbf{r}}}{\left[\left(\gamma_{20}+i\Delta_{1}\right)+\frac{G_{1}^{2}}{\Gamma_{21}\text{+}\Gamma_{20}}+\frac{G_{1}^{2}}{\gamma_{10}}+\frac{G_{2}^{2}}{\gamma_{10}}\right]}$ (S3d)

For the second step, the coupling equations are written as

(S4a)

(S4b)

(S4c)

Similarly to Eq. (S3d), we have

(S4d)

For the third step, by solving under steady state condition, we can obtain

(S5a)

Based on Eqs. (S3d), (S4d) and (S5a), the expression for the density matrix element of anti-Stokes FWM signal is obtained as

$\begin{matrix} \rho_{20(ASt)}^{(3)}=\frac{-iG_{2}^{\text{*}}G_{1}^{2}}{\left[\left(\gamma_{20}\text{+}i\Delta_{1}\right)+G_{1}^{2}/\left(\Gamma_{21}\text{+}\Gamma_{20}\right)+G_{1}^{2}/\gamma_{10}+G_{2}^{2}/\gamma_{10}\right]\left[\gamma_{20}+i\Delta'_{1}\right]} \\ \times\frac{1}{\left[\gamma_{10}+G_{1}^{2}/(\gamma_{20}+i\Delta_{1})+G_{1}^{2}/(\gamma_{12}-i\Delta_{1})+G_{2}^{2}/(\gamma_{20}+i\Delta_{1})\right]} \end{matrix}$ (S5b)

**2. The Third-Order Density Matrix element** $\rho_{21(St)}^{(3)}$

The density-matrix equations are given by

(S6a)

(S6b)

(S6c)

(S6d)

(S6e)

The ***E****_St_* signal can be described via perturbation . For the first process, a ground state ($\rho_{00}^{(0)}\text{+}\rho_{11}^{(0)}\text{=1}$) particle absorb a probe photon and transits to state$\rho_{21}^{(1)}$. With Eq. (S6a) solved under the weak field approximation ($\tilde{\rho}_{01}^{(r-1)}\approx\tilde{\rho}_{11}^{(r-1)}\approx\tilde{\rho}_{22}^{(r-1)}\approx0$) and the steady state approximation ($\partial\tilde{\rho}_{21}^{(r)}/\partial t=0$), we can obtain , which can also be equivalent to

(S7a)

Similarly, the second step expressed ascan be explained by solving Eq. (S6b) under weak field and steady state condition. As a result, we can obtain

(S7b)

For the third step expressed as, according to Eq. (S6a) under the approximation of weak field and steady state, we have

(S7c)

Based on Eq. (S7a), (S7b) and (S7c), we can finally get

(S7d)

With the dressing effects considered, the perturbation chain then can be modified as. During the first step, according to the *dressed perturbation chain* method and steady state approximation，we have

(S8a)

(S8b)

(S8c)

By solving Eqs. (S8a)-(S8c) with $\rho_{00}^{(0)}\approx1$，$\rho_{11}^{(0)}\approx0$and$\rho_{22}^{(0)}\approx0$taken into consideration，we have

(S8d)

For the second step, based on Eqs. (S8a), (S8b) and (S9a), we can obtain

(S9a)

As a consequence, $\tilde{\rho}_{01}^{(2)}$ is described as

(S9b)

The third step can be explained by

(S10a)

Consequently, $\tilde{\rho}_{21}^{(3)}$with approximation can be described as

(S10b)

Based on Eqs. (S8d), (S9b) and (S10b), the expression for the density matrix element $\rho_{21(St)}^{(3)}$can be given as

$\begin{matrix} \rho_{21(St)}^{(3)}=\frac{-iG_{c}^{*}G_{1}^{2}}{\left[\gamma_{21}+i\Delta'_{1}+G_{1}^{2}/\left(\Gamma_{20}\text{+}\Gamma_{21}\right)+G_{1}^{2}/\gamma_{01}+G_{\text{c}}^{2}/\gamma_{01}\right]\left[\gamma_{21}+i\Delta_{1}\right]} \\ \times\frac{1}{\left[\gamma_{01}+G_{1}^{2}/\left(\gamma_{21}+i\Delta'_{1}\right)+G_{1}^{2}/\left(\gamma_{02}-i\Delta_{1}\right)\right]} \end{matrix}$ (S10c)
